# Femmephobia Is a Uniquely Powerful Predictor of Anti-Gay Behavior

**DOI:** 10.1007/s10508-023-02704-5

**Published:** 2023-10-02

**Authors:** Rhea Ashley Hoskin, Karen L. Blair, Diane Holmberg

**Affiliations:** 1 Departments of Sociology & Legal Studies, Sexuality Marriage, & Family Studies, University of Waterloo and St. Jerome’s University, Waterloo, ON Canada; 2 Department of Psychology, Trent University, 1600 West Bank Drive, Peterborough, ON K9L OG2 Canada; 3 Department of Psychology, Acadia University, Wolfville, NS Canada

**Keywords:** Femmephobia, Homonegativity, Prejudice, Social Dominance, Femininity, Aggression

## Abstract

The current study explored a form of femmephobia (specifically, negative attitudes toward femininity in men) as a predictor of anti-gay behaviors among a sample of heterosexual men (*N* = 417). Additional predictor variables included hierarchical worldviews (i.e., social dominance orientation, right-wing authoritarianism, narcissism) and prejudicial attitudes (i.e., old-fashioned and modern homonegativity). Femmephobia emerged as a robust predictor, accounting for 23% of the variance in anti-gay behavior, surpassing the explanatory power (15%) of all other considered variables combined. Moreover, social dominance only predicted anti-gay behavior when femmephobia levels were high. Future research on discrimination and violence related to sexual identity and gender expression should incorporate femmephobia as a key predictive factor.

**Supplementary Information:**

The online version contains supplementary material available at 10.1007/s10508-023-02704-5.

## Introduction

In March 2013, David Beltier was walking with his boyfriend and their poodle Beauty, whose fur had been dyed pink. A man accosted the couple as they walked, shouting that their “poodle was a weird color and that’s just un-American” before physically assaulting Beltier (McDonough, 2013). Such anti-gay behavior is all too common, with a national probability sample suggesting approximately one in four sexual minority individuals in the USA have experienced actual or attempted physical attacks on their person or property motivated by their sexual identity, and almost 50% have been the targets of direct verbal abuse or harassment (Herek, 2009). Some theorists (e.g., see Morrison & Morrison, 2003) assume that anti-gay attitudes underlie such behavior, i.e., homophobia or homonegativity. Others (e.g., Altemeyer, 1998; Pratto et al., 1994) suggest that hierarchical worldviews, or beliefs that some groups, values, or individuals are inherently superior to others, are the driving motivation (e.g., “American” is superior to “un-American”). We agree that those theories provide part of the explanation for the attack on Beltier. However, we propose an additional contributing and understudied factor, namely femmephobia. We suggest that Beltier’s attacker may not have attacked solely based on Beltier and his partner being gay men but also (perhaps primarily) because Beauty’s pink fur contributed to the attacker’s perception of Beltier as a feminine man.

### Femmephobia

Femmephobia refers to the societal regulation and denigration of femininity across all gender and sexual identities, including inanimate objects perceived as feminine (Hoskin, 2017, 2020). Although biases against femininity have been acknowledged in particular instances, including sissyphobia (Bergling, 2001; Eguchi, 2011), trans-misogyny (Serano, 2007), anti-effeminacy (Sanchez & Vilain, 2012), and slut-shaming (Tanenbaum, 2015), to name a few (Hoskin, 2020), pulling them all together and recognizing the underlying current of femmephobia as a pervasive phenomenon occurred relatively recently (Hoskin, 2017, 2019, 2020). Femmephobic attitudes construe masculinity as superior to femininity, regardless of any individual’s gender, sex, or sexual orientation. While femininity is prescribed and tightly regulated for some, it can also be proscribed and seen as completely unacceptable for others. For example, femininity is viewed as being acceptable only in very specific circumstances (e.g., when enacted at just the right level and in the right contexts by straight cisgender able-bodied women), but not in any others. Theories invoking femmephobia are rich and have a multitude of implications for individuals of all gender and sexual identities (e.g., Blair & Hoskin, 2019; Bonnes, 2022; Davies, 2020; Hoskin & Serafini, 2023). In this paper, however, we focus on one specific prediction arising from this theory: discomfort with femininity in men will be a strong predictor of anti-gay behavior.^1^

Social discomfort with male femininity has been well-established (Glick et al., 2007; Jewell & Morrison, 2012; Serano, 2007). Individuals assigned male at birth who display feminine characteristics are at heightened risk of ridicule, bullying, peer rejection (Buijs et al., 2011; Kilianski, 2003; Taywaditep, 2001), social isolation, and criticism from peers and teachers (Bhana & Mayeza, 2019; Fagot, 1977; Harry, 1983; Matino & Cummings-Potvin, 2019). Much of this research has focused on children or adolescents. In adulthood, scholars have observed that straight men frequently define their masculine identity largely as being “not feminine” (Parrott et al., 2008; Sinacore et al., 2021; Thompson & Pleck, 1986). Rejection of feminine men, including feminine gay men, is thus theorized to be a means through which straight men can help define and reaffirm their own masculinity (e.g., Pascoe & Diefendorf, 2019; Theodore & Basow, 2000).

In line with these theories, masculine gender norms, including anti-femininity, are strongly associated with anger in response to gay men (Parrott et al., 2008). Such anger may fuel attacks. Men who self-report feminine behavior in childhood (D’haese et al., 2016) and adulthood (Dominic McCann et al., 2010; Sinacore et al., 2019; Woodford et al., 2013) report being targets of others’ anti-gay behaviors, including harassment and violence, at heightened rates.

The association between male femininity and anti-gay behavior is also reported by perpetrators. College students, particularly men, are more likely to self-report past anti-gay behaviors when they also endorse restrictive beliefs regrading when and for whom femininity and masculinity are appropriate (Franklin, 2000; Goodman & Moradi, 2008; Patel et al., 1995; Whitley, 2001). In experimental vignette research, straight men choose to avoid feminine-acting gay men more than masculine-acting ones (Schope & Eliason, 2004). Using qualitative methods, Buijs et al. (2011) found that male perpetrators of aggression against gay men frequently cited men’s feminine dress or behavior as instigating factors for aggression. Indeed, perpetrators often insisted they had no problem with homosexuality per se, only with feminine behavior in men. Given the strong theoretical foundation and (still somewhat limited) empirical work, our first hypothesis (H1) is that a discomfort with male femininity (i.e., one aspect of femmephobia) will be positively associated with straight men engaging in anti-gay behavior.

### Femmephobia in the Context of Other Predictors of Anti-Gay Behavior

Sinacore et al. (2021) argue that past research has largely conflated violence based on sexual orientation (i.e., one form of anti-gay behavior) with violence based on gender expression and that scholars must pay more attention to disentangling these two constructs. Further, Sinacore et al. point out that much of the existing research has focused on the experiences of children or adolescents, leaving experiences in adulthood relatively understudied. Therefore, it is important to explore the role of femmephobia as a predictor of anti-gay behavior not only on its own, but also in the context of other known predictors, to move further toward Sinacore et al.’s (2021) goal of disentangling gender expression from other established risk factors for anti-gay behavior.

#### Homonegativity

The most obvious candidate as a predictor of anti-gay behavior is homophobia or homonegativity, i.e., prejudicial attitudes toward individuals perceived as gay, lesbian, or in a same-sex relationship (McDermott & Blair, 2012). In the literature, two forms of homonegativity are proposed and assessed. Old-fashioned homophobia refers to “unfavorable social judgments about gay men and lesbian women attributable to a respondent’s moral convictions/biblical injunctions against homosexuality […] or beliefs that homosexuality should be considered some form of psychopathology” (McDermott & Blair, 2012, p. 2; Morrison et al., 2005, 2009). Those high in old-fashioned homophobia endorse items directly stating that homosexuality is morally wrong and worthy of condemnation.

Morrison et al. (2005) argue that as it has become less socially acceptable to hold such overt attitudes, the dominant mode of sexual prejudice has changed to a more covert and subtle form of prejudice, which they term modern homonegativity. Similar to other constructs, such as modern racism (McConahay, 1986), modern homonegativity does not directly assert that homosexuality is wrong; instead, the focus is on the belief that gay people are being inappropriately vocal in defense of their rights because they are no longer the target of discrimination.

Homonegative attitudes are associated with self-reported anti-gay behaviors (e.g., bullying, using homophobic language) in multiple large-scale surveys of adolescents (e.g., Poteat, 2008; Prati, 2012; Weber & Gredig, 2018). As Sinacore et al. (2021) noted, there is less work exploring these associations in adult samples, and none appear to use anything other than college/university students. Still, the pattern is similar: Homonegativity is associated with self-reported past physical and verbal anti-gay behavior in US college samples (Franklin, 2000; Goodman & Moradi, 2008; Patel et al., 1995; Schope & Eliason, 2000; Whitley, 2001). Small-scale laboratory studies have also shown homonegative attitudes predicting real-time negative behaviors toward confederates presumed to be gay, such as physical avoidance (Morrison & Morrison, 2003) and administration of higher levels of (supposed) electric shock (Bernat et al., 2001).

#### Hierarchical Worldviews

Past research has also suggested that discrimination, including anti-gay behavior, can be predicted by viewing the world as hierarchically organized. Altemeyer (1998) assessed a wide variety of attitudes, beliefs, values, and personality characteristics and found that two hierarchical worldviews, social dominance orientation (SDO) and right-wing authoritarianism (RWA), emerged as the strongest predictors of many prejudicial attitudes, including homonegativity.

Those high in SDO believe that society is fundamentally hierarchical and want to see themselves and their social group at the top of that hierarchy. Groups perceived as weak provide easy targets for domination and exertion of power (Altemeyer, 1998). This worldview predicts a wide variety of prejudices (Blair, 2017; Whitley, 1999), including homophobic attitudes (Adams et al., 2016; Nagoshi et al., 2008, 2019; Norton & Herek, 2013; Tee & Hegarty, 2006; Warriner et al., 2013; Willoughby et al., 2010). Work focusing on homophobic or anti-gay behaviors rather than attitudes is much rarer. However, higher levels of SDO were associated with self-reported past anti-gay behaviors in one study of university students (Goodman & Moradi, 2008).

RWA (Altemeyer, 1981, 1996, 2001) also concerns hierarchies, proposing that some individuals, beliefs, or practices are superior to others. Right-wing authoritarians see themselves as superior in being more moral and upstanding than most. Those high in RWA view ingroup authorities as fundamentally superior individuals worthy of unquestioned obedience. Traditional practices are seen as inherently superior to new or radical ideas. RWA is frequently associated with homophobic and transphobic attitudes (Adams et al., 2016; Nagoshi et al., 2008, 2019; Norton & Herek, 2013; Tee & Hegarty, 2006; Warriner et al., 2013; Willoughby et al., 2010). Again, work focusing on anti-gay behaviors rather than attitudes is much scarcer. However, Goodman and Moradi (2008) found that RWA was associated with more self-reported past anti-gay behaviors and fewer pro-gay behaviors in a university sample. Those high in RWA are also favorable toward anti-gay behaviors in principle. They are more likely than the general population to approve of “gay-bashing” (Altemeyer, 1996, 2001) and are more inclined than those lower in RWA to say they would “help the government if it set out to harass, imprison, torture and even execute gays” (Altemeyer, 1988, p. 116).

Finally, narcissism applies a hierarchical worldview to the self. Individuals with narcissistic characteristics see themselves as inherently superior to others, deserving special treatment. Although not examined as extensively as the other two worldviews, high narcissism is an established predictor of general anger, aggression (e.g., Lambe et al., 2018; Papps & O’Carroll, 1998), and dehumanization of others (Lock, 2009). Narcissism has received little attention as a predictor of anti-gay behaviors specifically; however, narcissism is associated with other types of gender-based violence, including domestic violence and violence against women (Altemeyer & Hunsberger, 1992, as cited in de Zavala et al., 2021). Further, “national collective narcissism” (i.e., the belief that “one’s group is exceptional but not sufficiently recognized by others”; de Zavala et al., 2021, p. 2) predicts self-reported homophobia, sexism, and intergroup hostility (de Zavala et al., 2021; Mole et al., 2021). Narcissism is, therefore, an underexplored but theoretically relevant predictor of anti-gay behavior.

Thus, these variables have individually predicted anti-gay behavior in past research. To our knowledge, though, very little past research has heeded Sinacore et al.’s (2021) call and explored femmephobia, specifically discomfort with male femininity, as a unique predictor of anti-gay behavior, able to account for variability in anti-gay behaviors even when controlling for other predictors. In fact, we were only able to find two examples. First, Woodford et al. (2012) found that discomfort with male femininity uniquely predicted the use of “that’s so gay” to indicate something was odd or undesirable (i.e., a homonegative micro-aggression), even when other more traditional indicators of anti-gay attitudes (e.g., beliefs regarding LGBTQ + access to relationships and employment rights) did not.

Second, Goodman and Moradi (2008) found that in university students, endorsement of traditional gender roles (a construct that is related to, yet distinct from, femmephobia; see Discussion) was a strong predictor of self-reported past anti-gay behaviors and remained so even when included in the same model as old-fashioned homonegativity, SDO, and RWA. Goodman and Moradi (2008) note that their findings suggest that attitudes surrounding gender are an important, unique, and understudied construct to consider when studying anti-gay behavior.

Our study will extend Goodman and Moradi’s (2008) work by considering these issues in a non-university sample, adding new potential predictors of anti-gay behaviors, and focusing squarely on femmephobia rather than the more diffuse construct of traditional gender-role attitudes. In line with past research, we expect that each of the measures reviewed (i.e., old-fashioned homonegativity, modern homonegativity, SDO, RWA, and narcissism) will be positively associated with self-reported anti-gay behavior; however, in line with Goodman and Moradi’s (2008) findings, our second hypothesis (H2) is that femmephobia will remain a significant predictor of anti-gay behavior, even when controlling for these other established predictors.

### Femmephobia as a Potential Moderator of the Other Predictors

The final issue we will explore is whether femmephobia might serve as a moderator, strengthening the association between the other constructs and anti-gay behavior. As the Goodman and Moradi (2008) paper did not consider these potential interactive effects, this represents an additional manner in which our work extends theirs.

Old-fashioned homonegativity views homosexuality as morally wrong; modern homonegativity views gay individuals as pushing too hard to achieve the rights they (presumably) already have. Taken at face value, neither of those views would necessarily drive anti-gay behaviors such as ostracism or violence. Instead, one could try to convert the “morally misguided person” (“hate the sin, love the sinner”) or educate them regarding their existing rights (and a lack of need for “special rights”). Matsumoto et al. (2014) show that simple emotions are stronger predictors of behavior than complex attitudes. Perhaps homonegativity primarily gets translated into actual anti-gay behavior when paired with the more visceral sense of disgust elicited by viewing misplaced femininity.

Likewise, hierarchical worldviews dictate that some groups, beliefs, or individuals are superior to others, but they do not necessarily mark out specific targets as inferior. Again, the links between hierarchical worldviews and anti-gay behavior may be particularly strong for those high in femmephobia, who may perceive gay individuals (particularly feminine gay men) as inferior. After all, de Zavala et al. (2021) suggest that outgroups are seen as particularly problematic when they threaten the purity of a valued identity. When individuals view outgroups as immoral or impure, prejudicial attitudes are likely to shift from anger to actual aggression (Frank et al., 2015; Matsumoto et al., 2014). Femmephobia may be a driving force that helps identify those who exhibit misplaced femininity as immoral or impure, thus meriting aggression and other negative behaviors. Thus, our third hypothesis (H3) is that there will be moderation effects, such that the association between the other predictor variables and anti-gay behavior will be stronger when femmephobia is high than when it is low.

### Current Study

Thus, we have proposed three overall hypotheses:

H1: Femmephobia will be positively associated with straight men engaging in anti-gay behavior.

H2: Femmephobia will remain a significant predictor of anti-gay behavior, even when controlling for modern and old-fashioned homophobia/homonegativity, social dominance orientation, right-wing authoritarianism, and narcissism.

H3: There will be moderation effects, such that the association between our other predictors (see H2) and anti-gay behavior will be stronger when femmephobia is high than when it is low.

To test these hypotheses, we asked a group of straight, cisgender men to complete an online survey. We assessed whether femmephobia predicted self-reported past anti-gay behaviors independently and when considered with the other measures. We also assessed whether femmephobia interacted with each of the other measures, expecting that the association between the other measure and anti-gay behavior would be stronger when femmephobia was high than when it was low.

## Method

### Participants

A total of 554 participants completed the online survey. To be included in the current study, participants (1) had to indicate their gender was “male” (*sic*), or (2) if they indicated their gender was “other,” they had to choose “Cis Man” from a drop-down list of gender identities, and (3) they had to indicate their sexual identity was “straight/heterosexual.” Participants also needed complete responses on all the measures in the current study. Although recruitment material was aimed at men aged 18–35, as they were the focus of the follow-up laboratory study, older men were not excluded. The final sample size for the current study was 417. Participants excluded from the current study’s sample did not differ from those included on the main outcome variable or on any demographic variables.

The average participant age was 28.1 (range, 18 to 65, *SD* = 7.5). Participants reported an average of 14.8 years of formal education (*SD* = 2.38) and were predominantly from the USA (91.1%). The majority of participants identified their ethnicity as White (87.3%), followed by Hispanic (5.1%), other/mixed-race (3.9%), Asian (2%), Native American (1%), or Black (0.7%).

### Procedure

The research team circulated recruitment materials through Facebook advertisements, postcard mailings, and flyers distributed in areas such as parking lots and local establishments, inviting men aged 18–35 to participate in a study of “attitudes and opinions.” After providing consent, participants completed an online survey consisting of measures relating to a wide variety of prejudices, such as Islamophobia, racism, sexism, homophobia, and transphobia. The current study’s measures were embedded within these other measures; there was no mention of the study’s focus on issues surrounding sexual identity and homophobia. This online study served as a screening tool for a follow-up laboratory study of the physiology of sexual prejudice (O’Handley et al., 2017). All measures for the current paper come from the initial online survey.

### Measures

#### Self-Report of Behavior Scale

The dependent variable, anti-gay behavior, was assessed using Roderick et al.’s (1998) revised version of the Self-Report of Behavior Scale (SBS-R), which they designed to identify behavioral manifestations of homonegativity toward both gay men and lesbians. This scale consists of 20 items rated on a five-point Likert-type scale ranging from 1 (*never*) to 5 (*always*). Items ranged from avoidance behaviors (e.g., “When a gay person has been near me, I have moved away to put more distance between us”), through negative verbal behavior (e.g., “I have spread negative talk about someone because I suspected that he or she was gay”), to physical assault (e.g., “I have gotten into a physical fight with a gay person because I thought he or she had been making moves on me”). Roderick et al. (1998) found good internal consistency (Cronbach’s alpha = 0.92) and little association with a measure of social desirability.

Following Roderick et al. (1998), we calculated total scores by summing the individual items. One of the 20 items was inadvertently omitted from our survey, leaving 19 items, with a possible range of 19 to 95. Twenty-eight participants completed most items on the scale but had missing data for either one or two items. We used simple imputation to fill in these missing scores. Cronbach’s alpha for the scale in this study was 0.91.

#### Femmephobia

The current study provisionally assessed one aspect of the broader construct of femmephobia, specifically discomfort with male femininity. We assessed this construct using a modified version of Hill and Willoughby’s (2005) genderism and transphobia measure, created for the current study (see Supplemental Materials). It consisted of nine items, each rated on a 7-point scale ranging from 1 (*strongly disagree*) to 7 (*strongly agree*). A sample item is “Men who act like women should be ashamed of themselves.” Cronbach’s alpha in this study was 0.90.

#### Homonegativity

Participants completed two validated measures of homonegativity. The attitudes toward gay men subscale of the Attitudes toward Lesbian and Gay Men Scale (ATG; Herek, 1994) assesses old-fashioned homonegativity directed toward gay men. These items measure explicit attitudes that male homosexuality is unacceptable and that gay men deserve fewer rights than straight men. A sample item is “Male homosexuality is a perversion.” The scale consists of 10 items rated on a 5-point Likert-type scale, ranging from 1 (*strongly disagree*) to 5 (*strongly agree*). Cronbach’s alpha in previous research was 0.89, and the scale demonstrated good convergent validity with other theoretically relevant measures (Herek, 1988). Cronbach’s alpha in the current sample was 0.95.

The gay male version of the Modern Homonegativity Scale (MHS-G; Morrison & Morrison, 2003; Morrison et al., 2009) assesses more modern or subtle forms of prejudice toward gay men. Items focus on ideas like gay men are pushing too hard for special privileges that they already have all the rights they need, and that specifically focusing on or celebrating gay issues is inappropriate. A sample item is “Gay men should stop complaining about the way they are treated in society, and simply get on with their lives.” The scale consists of 10 items answered on a 5-point Likert-type scale, ranging from 1 (s*trongly disagree*) to 5 (*strongly agree*). In previous research, the measure has been unidimensional, reliable, and distinct from old-fashioned homonegativity (Morrison & Morrison, 2003). Cronbach’s alpha in the current sample was 0.94.

#### Hierarchical Worldviews

Participants completed three measures assessing hierarchical worldviews theoretically and empirically related to prejudice in past research. The Social Dominance Orientation Scale (SDO; Pratto et al., 1994) assesses a view that the world is essentially structured hierarchically rather than in an egalitarian fashion, with some groups being superior to others. A sample item is “Some groups of people are just inferior to other groups.” The measure consists of 16 items rated on a 7-point Likert-type scale, ranging from 1 (*very negative*) to 7 (*very positive*). The measure has been reliable and valid in previous research (Pratto et al., 1994). Cronbach’s alpha for the scale in this study was 0.90.

The Right Wing Authoritarianism Scale (RWAS; Altemeyer, 1988) assesses authoritarian views, i.e., advocating traditional values, adherence to law and order, and an urge to punish those who go against social norms. A sample item is “Our country will be destroyed someday if we do not smash the perversions eating away at our moral fiber and traditional beliefs.” The scale consists of 30 items to which participants respond using a 9-point Likert-type scale, ranging from v*ery strongly disagree* (-4) to *very strongly agree* (4). The scale has good reliability (Awad & Hall-Clark, 2009) and strong convergent validity with other measures of authoritarian personality (Goodman & Moradi, 2008). Cronbach’s alpha for the scale in this study was 0.97.

We measured narcissism using the Narcissistic Personality Inventory or NPI-16 (Ames et al., 2006), a shorter version of Raskin and Terry’s (1988) NPI-40. The NPI-16 consists of 16 dichotomous item pairs labeled A and B. Participants must select the statement that best describes them, for example, “A: I am no better or worse than most people.” versus “B: I think I am a special person.” The final score is a total across all items assessing a narcissistic worldview, i.e., a belief that one is superior to others and inherently deserves special treatment. The measure shows adequate reliability and good convergent and divergent validity (Gentile et al., 2013). Cronbach’s alpha for the scale in this study was 0.72.

## Results

Table 1 presents descriptive statistics for each measure and correlations between all study variables. Means for each variable were at or somewhat below the scale midpoint, suggesting only moderate levels of each construct on average, but there was substantial range/variability on each measure. Consistent with expectations, each predictor variable was significantly and positively associated with the outcome variable (column 1). Predictor variables were also strongly correlated with each other, except for narcissism, which showed only weak associations with the other constructs.

**Table 1:** Correlation matrix and descriptive statistics for all study variables

	Variable	1	2	3	4	5	6	7	M	SD	Observed Range
1	Anti-gay behavior	–							22.27	6.18	19–62
2	Modern homonegativity	.28^*^	–						3.07	1.05	1–5
3	Old-fashioned homonegativity	.26^*^	.77^*^	–					2.68	1.16	1–5
4	Social dominance orientation	.35^*^	.61^*^	.48^*^	–				2.75	1.04	1–6.25
5	Right-wing authoritarianism	.23^*^	.74^*^	.82^*^	.45^*^	–			− 0.59	1.63	− 3.97–2.74
6	Narcissism	.15‡	.12†	.03	.14^‡^	.01	–		5.61	3.22	0–15
7	Femmephobia	.48^*^	.68^*^	.71^*^	.54^*^	.63^*^	.11^†^	–	2.79	1.27	1–6.9
	Possible Range	19–95	1–5	1–5	1–7	− 4– + 4	0–16	1–7			

^†^*p* < .05, ^‡^*p* < .01, *p < .001

The main analyses consisted of regression analyses conducted in SPSS v28. All predictor variables were mean-centered prior to analysis and the creation of interaction terms. The predictor variables were somewhat positively skewed, and the dependent variable was very positively skewed, potentially violating multivariate normality and homoscedasticity assumptions and potentially affecting *p* values. Therefore, we employed bootstrapping for all analyses, which does not hold the same assumptions and is appropriate for skewed data (Hayes, 2018). Bootstrapping involves repeating the analysis in many random samples (1000 in this case), drawn randomly with replacement from the study sample. Results are shown as 95% confidence intervals around the unstandardized regression coefficients (i.e., *b*s). Confidence intervals that do not cross zero correspond to significant effects at *p* < 0.05. These effects are bolded in the table.

Note that bootstrapping only provides bootstrapped *b* coefficients; it does not provide bootstrapped versions of standardized coefficients (i.e., betas) or of *R*^2^ statistics. To provide full information and context for readers more familiar with non-bootstrapped regressions, the betas and *R*^2^ values are reported in the tables, taken from the original non-bootstrapped analyses; however, the interpretation of findings rests on the bootstrapped results.

To test H1, a simple regression analysis was conducted, with femmephobia as the predictor variable and anti-gay behavior as the dependent variable. As predicted, femmephobia was a strong and significant positive predictor of engaging in more anti-gay behavior, β = 0.48, *b* = 0.12, *SE* = 0.02, 95% bootstrapped confidence interval: [0.10, 0.16], *R*^2^ = 0.23, *p* < 0.001.

To test H2, a hierarchical regression analysis was conducted to assess whether femmephobia continued to be a significant predictor of anti-gay behavior, over and above the other more traditional predictors of prejudice. Table 2 (first column) shows that when all five traditional predictors of prejudice were considered together,^2^ only social dominance orientation retained its significance. In the next step (second column), femmephobia was added, and was a significant predictor. In fact, femmephobia was even more strongly associated with the outcome variable than when considered on its own, even when competing for variance with five other predictor variables. Furthermore, femmephobia accounted for as much variance by itself as did the other five predictors put together.

**Table 2:** Femmephobia as a predictor of anti-gay behavior, in conjunction with all other study variables, main effects plus interactions

	Initial			Adding femmephobia			Adding interactions		
Variable	β	*b* (*SE*)	95% CI	β	*b* (*SE*)	95% CI	β	*b* (*SE*)	95% CI
Modern homonegativity	.03	.01 (.03)	[− .04, .07]	− .06	− .02 (.03)	[− .07, .04]	− .02	− .01 (.03)	[− .06, .05]
Old-fashioned homonegativity	.13	.04 (.03)	[− .01, .09]	− .11	− .03 (.02)	[− .08, .01]	** − .19**	** − .05 (.02)**	**[− .10, − .01]**
Social dominance orientation	**.26**	**.08 (.02)**	**[.04, .12]**	**.16**	**.05 (.02)**	**[.02, .08]**	**.12**	**.04 (.01)**	**[.01, .06]**
Right- Wing authoritarianism	− .02	− .00 (.02)	[− .03, .03]	− .06	− .01 (.02)	[− .04, .02]	.07	.01 (.02)	[− .02, .05]
Narcissism	.12	.01 (.01)	[− .00, .02]	.09	.01 (.01)	[− .00, .02]	.08	.01 (.01)	[− .00, .02]
*R*^2^	.15^*^								
Femmephobia				**.55**	**.14 (.02)**	**[.10, .18]**	**.44**	**.11 (.02)**	**[.07, .15]**
Δ* R*^2^				.13*					
Femme. X modern HN							** − .19**	** − .04 (.02)**	**[− .10, − .01]**
Femme. X Old-fashioned HN							.09	.02 (.03)	[− .02, .08]
Femme. X social dominance							**.29**	**.05 (.01)**	**[.03, .08]**
Femme. X right-wing auth							.11	.02 (.01)	[− .01, .04]
Femme. X narcissism							.15	.01 (.01)	[− .00, .02]
Δ* R*^2^							.10*		
Final *R*^2^							.38*		

*Femme.* Femmephobia, *HN* Homonegativity, *Auth.* Authoritarianism

95% CI = 95% bootstrapped confidence interval

*p < .001

To test H3, interactions involving femmephobia were added (third column). When the interactions were added, femmephobia and social dominance orientation remained as positive and significant predictors of anti-gay behavior. Unexpectedly, however, old-fashioned homonegativity was now significantly negatively, not positively, associated with anti-gay behaviors.^3^ Simple slopes analyses revealed that when femmephobia was low (1 SD below the mean), the association between social dominance orientation and anti-gay behavior was not significant at all (*b* = -0.64, *SE* = 0.42, *p* = 0.13). However, when femmephobia was high (1 SD above the mean), the association between social dominance orientation and anti-gay behavior was positive and statistically significant (*b* = 1.96, *SE* = 0.36, *p* < 0.001).

Contrary to expectations, modern homonegativity showed a reversal of this pattern (see Figure 2). When femmephobia was low, the association between modern homonegativity and anti-gay behavior was not significant (*b* = 0.88, *SE* = 0.53, *p* = 0.10). When femmephobia was high, the association between modern homonegativity and anti-gay behavior was significant but was, unexpectedly, negative rather than positive (*b* = -1.05, *SE* = 0.53, *p* = 0.05).^4^

## Discussion

### Femmephobia as a Uniquely Powerful Predictor of Anti-Gay Behavior

Femmephobia far exceeded our expectations as a predictor of anti-gay behavior. Not only was it a strong predictor on its own, supporting H1, but it also retained its strong predictive power over and above the more traditional predictors, such as homonegativity and hierarchical worldviews (H2). In fact, femmephobia was a stronger predictor of anti-gay behaviors than all the other predictor variables combined. As can be seen in Table 2, the other five predictor variables combined accounted for 15% of the variability in the measure of anti-gay behavior. Femmephobia accounted for 23% of the variability when considered on its own (see simple regression). It still added 13% additional variance over and above the other five predictors, plus another 10% when it was considered in interactions with the other variables. If one wanted to pick only one construct as the strongest predictor of anti-gay behaviors, perhaps as a potential intervention target, femmephobia would be the optimal choice.

Our findings regarding the key role of femmephobia partially parallel the findings of Goodman and Moradi (2008). They too found that a gender-related measure, specifically one assessing endorsement of traditional gender roles, was a strong predictor of anti-gay behaviors; however, our measure of femmephobia performed even better than their gender-role measure. These differences might potentially be attributable to other differences between the two studies (e.g., sample characteristics, analysis strategies, inclusion of other predictor variables). However, we suspect that the primary reason our measure outperformed Goodman and Moradi’s (2008) was that, along with others (e.g., Whitley & Ægisdóttir, 2000), they may have slightly missed the mark by focusing on general cognitively grounded gender-role beliefs, rather than on femmephobia specifically. As Hoskin (2020) notes, gender is not simply “divided” by roles; instead, there is a perceived hierarchy, with masculinity consistently being viewed as superior to femininity. People do not simply hold abstract, non-emotional views about appropriate gender roles; instead, many have strong, visceral, emotional, and morally outraged reactions to the presence of “misplaced” femininity. We concur with other researchers (e.g., Matsumoto et al., 2014) that strong emotions and perceptions of a group as immoral are the most potent catalysts for prejudiced behaviors. For many, femmephobia provides precisely this sort of motivational impetus.

### Femmephobia’s Role in Conjunction with Other Predictors

In addition to being the single strongest predictor of anti-gay behaviors in our study, femmephobia also acted in unexpected ways when considered in conjunction with the other study variables. The other five predictor variables performed as typically seen when proceeding from bivariate to multivariate analyses, e.g., from correlations to multiple regressions. For example, each variable was strongly and significantly associated with the outcome variable when considered on its own (see Table 1), but when they were all considered simultaneously in a single regression equation (Table 2, first column), each one’s predictive power dropped substantially, leaving only SDO as a significant predictor of anti-gay behaviors, at a reduced strength. This pattern occurs because multiple regression analyses remove the overlapping variance from predictor variables, leaving only the unique predictive power of each variable represented in the *b*s and betas. Typically, as one adds more and more related predictor variables, the *b*s and betas for each one tend to get smaller, as it becomes harder for each variable to add a unique piece to the puzzle, something that no other variable or combination of variables can explain.

Femmephobia, however, did not show that typical pattern. Unusually, its *b* and beta grew somewhat stronger, not weaker, when it was considered alongside the other variables (β = 0.48, *b* = 0.12, when considered on its own; β = 0.55, *b* = 0.14 when added to the other five predictors). This pattern indicates the presence of a suppressor effect (see Hodson & Prusaczyk, 2023, for a discussion), which occurs when two or more predictor variables are correlated with each other, at least in part, for reasons that do not have very much to do with their association with the outcome variable. When that unrelated variance is suppressed or set aside by removing the shared variance between the predictor variables, it can, at times, allow the unique effects of one of the variables to shine through even more clearly (Pandey & Elliott, 2010).

Here, femmephobia is positively related to each of the other predictor variables, often very strongly (see bottom row, Table 1). Those who are femmephobic also tend to be homophobic, authoritarian, narcissistic, and generally prejudiced in all the traditional ways. But despite its strong associations with the other predictors, femmephobia is not merely one more predictor variable, similar to all the others. Instead, it brings new and different information to the table. That piece of the puzzle unique to femmephobia, i.e., disgust and denigration in the face of misplaced femininity, remains a very strong predictor of anti-gay behavior, even when all of its overlap with other predictor variables is removed.

**Fig. 1:**
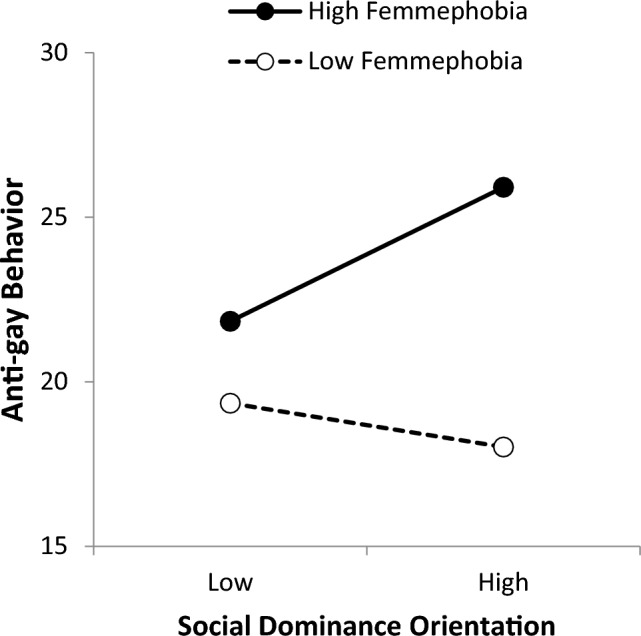
Social dominance orientation by femmephobia interaction. *Note:* Graph is shown at one standard deviation above and below the mean of each variable

Furthermore, as suspected, femmephobia seems to work in concert with some of the other variables to accentuate their effects. Social dominance orientation retains its predictive power even when all other variables are controlled for. Still, as shown in Fig. 1, social dominance does not translate into anti-gay behaviors without femmephobia also being present. Femmephobia may mark gay people (with their “misplaced” or “unregulated” ways of displaying femininity) as “inferior” individuals over whom social dominance is appropriately exercised. Although the interactions missed statistical significance when bootstrapping was used, right-wing authoritarianism and narcissism showed identical patterns. Thus, femmephobia appears to be a key ingredient that marks gay people as “inferior” and worthy of negative behaviors in the eyes of those with hierarchical worldviews.

Unexpectedly, however, modern homonegativity showed a very different pattern. Old-fashioned homonegativity also showed a puzzling reversal, switching its sign from positive to negative whenever femmephobia was introduced into the equation. Why? Again, these patterns can be attributed to suppressor effects, in which the meaning or interpretation of one variable can change when overlapping variance with another variable in the equation is removed. One possibility is that once the basic overlapping variance with femmephobia is removed (i.e., basic homonegativity, viewing gay individuals as inferior to straight individuals), what is left over in “old-fashioned” homonegativity is simply a general tendency to be old-fashioned.^5^ In line with this supposition, when we controlled for age and religiosity in exploratory analyses, the puzzling negative association between old-fashioned homonegativity and anti-gay behavior disappeared, replaced by a null association.

**Fig. 2:**
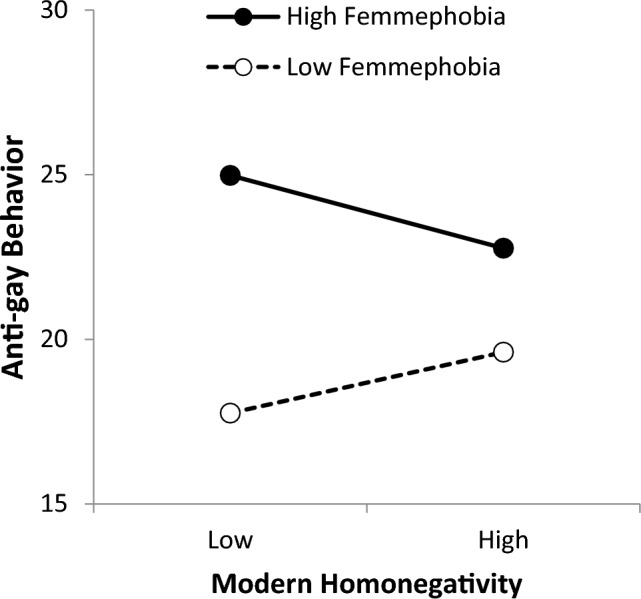
Modern homonegativity by femmephobia interaction. *Note:* Graph is shown at one standard deviation above and below the mean of each variable

A similar approach might explain the unexpected negative association between modern homonegativity and anti-gay behavior at high levels of femmephobia (Fig. 2). What remains if core homonegativity is removed from the modern homonegativity measure by removing overlapping variance with femmephobia? A review of the items suggests that what remains is a general belief that no one should ever get any special treatment; everyone should always expect to be treated the same as everyone else. Those high in femmephobia may still feel negatively toward gay people. However, as their commitment to this perspective of “no differential treatment for anyone, for good or ill” increases, they may work especially hard to keep their negative emotions from turning into actual negative behavior.

Again, these interpretations of the unexpected findings are speculative.^6^ They serve as a reminder that when one includes multiple predictor variables in a regression, the meaning of the unique predictive association of each variable with the outcome measure can stray further and further from the basic bivariate associations. Although statistically interesting, it does not always make much practical sense to interpret the residualized, unique aspects of each measure, freed from overlap with all other measures. In the real world, these constructs all tend to be positively related, all complementing and reinforcing each other, rather than being pitted against each other to remove any overlapping association (see Hodson & Prusaczyk, 2023, for a similar call for caution when interpreting suppressor effects).

Returning then to the simplicity of the correlation matrix in Table 1, we see that every single one of the constructs we assessed was significantly and positively associated with engaging in more anti-gay behavior, as expected. However, femmephobia was the single strongest individual predictor, and the regression analyses show us that it tells a uniquely important part of the story. By itself, femmephobia accounted for more variance than all the other variables combined. Considered in association with the other predictor variables in interactions, it added even more explanatory power. Femmephobia is a formidable and understudied factor that deserves much deeper research attention.

### Strengths and Limitations

The current study has many strengths. It includes two understudied predictors of anti-gay behavior, i.e., femmephobia and narcissism, and identifies femmephobia as a uniquely powerful predictor. Unlike most research in the field, it focuses on behavioral outcomes (rather than attitudes). It uses a relatively large non-student adult sample and focuses on straight, cisgender men, the group most likely to engage in anti-gay behaviors in everyday life (Kite et al., 2021).

However, there are also several important limitations to consider. First, as already noted, there was a slight disconnect between our primary predictor variable (focusing on feminine men) and the outcome variable, which assessed behavior toward both gay men and lesbians (notably, while excluding bisexual and other sexual minority individuals completely). While this disconnect regarding target groups across measures was certainly not ideal, it only works against our hypotheses, making our tests, if anything, conservative.

Second, the assessment of femmephobia in the current study (i.e., focusing on straight cisgender men’s views of feminine men) was preliminary and incomplete. Femmephobia is a complex attitude held by many, both within and outside of LGBTQ + communities, and can involve many targets beyond feminine men. For example, gay men express femmephobia to each other when they label their Grindr profiles with “no femmes” (Taylor & Hoskin, 2023). The queer community frequently questions the authenticity of femme lesbians’ sexual identities (Blair & Hoskin, 2015, 2016). Feminine lesbians may be more likely to experience sexual harassment or the fetishization of their relationships by outsiders, potentially contributing to experiences of sexual violence (see Matheson et al., 2021). A more complex and multifaceted measure that can assess the many aspects of femmephobia is required and is currently under development.

Third, our study relies on self-report, which always has the potential to be affected by social desirability. Nonetheless, our findings held up well when we controlled for social desirability; furthermore, self-report remains one of the best predictors of actual future behavior (Carnaghi et al., 2007), and the anonymous, online nature of the survey likely served to reduce the salience of social desirability cues.

Finally, our correlational study means we cannot assume femmephobic attitudes are necessarily the cause of anti-gay behaviors; reverse causation or third variables are always possible. While observational and experimental studies in this area to help establish causality would be desirable, it is challenging to design ecologically valid, yet still ethically permissible, experimental studies involving prejudice and aggression. Furthermore, it is difficult to randomly assign participants to meaningful variations in well-entrenched attitudes such as femmephobia, nor is it particularly ethical to attempt to randomly assign people who do not already do so to hold femmephobic beliefs. Instead, we believe that future research should consider interventions to reduce femmephobia, compared with a wait-list control group, as an ethical means of assessing whether reduced femmephobia is causally related to reduced negative outcomes. Femmephobia is a uniquely powerful predictor of anti-gay behavior. If it should also prove to be a uniquely powerful *cause* of such behaviors, then it should prove to be a particularly fruitful avenue for future interventions and perhaps one more amenable to change than personality constructs or broad worldviews.

### Implications and Applications

What form might such interventions take? One possibility is intervening with children; after all, research suggests children (both boys and girls) are socialized from an early age to perceive rigid gender binaries and to value masculine attributes over feminine attributes (i.e., femmephobia; see Hoskin, 2017; Theodore & Basow, 2000). For example, in one study (Kreiger & Kochenderfer-Ladd, 2013), fourth-grade boys *and* girls who engaged in activities perceived as feminine experienced more victimization and less peer acceptance than those engaging in masculine activities. Hoskin and Serafini (2023) suggest that femmephobic attitudes develop early in life and that social institutions and systems, such as the family, school, popular culture, religion, and sports, further solidify these attitudes. Fortunately, each context also represents a place where such messages could be “unlearned.” Psychoeducation alongside workshops and training for teachers, coaches, and parents could challenge the femmephobic messages children receive from society (e.g., *The Femmephobia 101 Workbook*, Hoskin et al., 2023).

Similarly, in adult populations, femmephobic attitudes could be unlearned through improved cultural messaging, exposure to diverse individuals comfortable with their femininity, and/or individual or group interventions. As one example, cognitive behavioral therapy (CBT) shows that changes in thinking and understanding can result in changes in behavior (Fenn & Byrne, 2013; Høifødt et al., 2011). Researchers and clinical practitioners should consider CBT-inspired interventions at the individual or group level to help individuals unlearn femmephobic attitudes and replace them with an appreciation of the advantages of both “masculine” and “feminine” characteristics, removed from their associations with sex and gender.

Strategies aimed at mitigating femmephobia could benefit not only the recipients of such prejudice but also those harboring femmephobic views. For instance, certain men’s unease with femininity has been linked to maladaptive social relationships throughout their lives (Pollitt et al., 2023). If society labels attributes such as nurturance, gentleness, empathy, emotional intimacy, and supportive touch as feminine, then the construction of masculinity becomes an act of refusal and repudiation of these qualities, potentially compromising men’s mental and physical health (Courtice et al., 2023). Clinicians and educators could facilitate men’s exploration of feminine traits, appreciation of femininity in others, and normalization of feminine expressions among their peers. If societal norms permitted individuals to freely embrace and express their femininity and masculinity as per their preference, it would enrich the range of emotional and social resources available to everyone.

### Conclusion

Our findings emphasize that negative views toward femininity in men powerfully predict anti-gay behavior, outstripping many other previously studied factors associated with anti-gay aggression and discrimination. The findings suggest that when understanding anti-gay behavior, assessing attitudes toward gender and gender expression, particularly femininity, is an important piece. Increasingly positive attitudes toward same-sex relationships, parenting, and the general inclusion of sexual minorities within society may be proliferating. However, ingrained societal notions about the “proper” displays of femininity and acceptable reactions to violations of norms surrounding femininity may persist. These enduring perceptions might explain why anti-gay behavior remains prominent even though societal acceptance of same-sex relationships, in principle, is at an all-time high.

Future research should continue to explore the systemic and automatic ways society devalues, denigrates, and regulates femininity. While our study concentrated on the actions of heterosexual men toward presumably gay men, the societal implications of femmephobia affect individuals across all genders and sexual orientations. The denigration of femininity that tacitly permits the ridiculing of a gay man walking a pink poodle also surfaces in the advice given to women in STEM to shun feminine expressions if they want to be perceived as competent (Bergsieker et al., 2021). Consequently, tackling the genesis and spread of femmephobic attitudes within society might present an innovative pathway to combat not only discrimination, harassment, and violence directed at sexual minorities, but also a broad array of societal problems arising from the normalized subjugation of femininity to masculinity.

### Supplementary Information

Below is the link to the electronic supplementary material.

Supplementary file1 (DOCX 21 kb)

## Data Availability

Data are not available to be shared publicly but may be available from the second author pending approval of the associated IRBs. Supplemental Materials for the manuscript provide a copy of the measure constructed for the current study. All other study materials are available from the sources cited in the methods section of the manuscript.

## Footnotes

1 The measures for the current paper came from a larger study assessing prejudice and discrimination against various groups. The available measure of anti-gay behavior included a variety of actions against gay people: ostracism, verbal abuse, property crimes, and threatened or actual physical violence. Our predictor variables focused on men or gay men specifically, but the available outcome variable of anti-gay behavior focused on gay *people*, which could in principle include both gay men and lesbians. We strongly suspect our participants (all straight men) primarily had gay men in mind when completing this measure. For example, several items assessed participants’ past reactions when a gay person supposedly hit on them; for straight men, such behavior would come from a gay man. We refer to our outcome variable throughout as “anti-gay behavior,” and acknowledge that it could encompass actions against both gay men and lesbians. However, we still see past research on discrimination against gay men as being especially relevant, given our sample and the research context.

2 The Supplemental Materials show results of additional analyses, adding femmephobia to the two sets of predictor variables separately.

3 In the non-bootstrapped analyses, the interaction between narcissism and femmephobia was also significant at *p* < .001, and the interaction between right-wing authoritarianism and femmephobia was marginally significant at *p* = .09. Both showed patterns identical to this one.

4 On an exploratory basis, we also repeated our analyses controlling for background variables related to prejudice in past research, namely age, education, religiosity, social desirability (specifically in the area of cultural sensitivity), and amount of contact with gay men. Results were very similar to those shown here: in the final analysis, femmephobia was still a strong and significant positive predictor of anti-gay behavior, and the same two interactions were statistically significant, showing the same patterns. The only difference was that neither social dominance orientation nor old-fashioned homonegativity were statistically significant predictors of anti-gay behavior when the covariates were included.

5 Note that Goodman and Moradi (2008), in their mediational model, found a somewhat similar finding for RWA. The indirect effect of RWA through old-fashioned homophobia was positive, but the residual direct effect when all other variables were controlled was, unexpectedly, negative. Their speculative explanations are similar to ours.

6 Note that Goodman and Moradi (2008) saw similar reversals of signs in their study, and offered similar tentative explanations.
