# Supplementary material for: Femmephobia Is a Uniquely Powerful Predictor of Anti-Gay Behavior
Source: Arch Sex Behav. 2023 Oct 2;53(1):127–40. doi: 10.1007/s10508-023-02704-5 (PMC10794376; doi:10.1007/s10508-023-02704-5)
Supplement: Supplementary file 1 — Supplementary file1 (DOCX 21 kb) [file 10508_2023_2704_MOESM1_ESM.docx]

**Femmephobia Is a Uniquely Powerful Predictor of Anti-Gay Behavior**

**Supplemental Material**

**Section 1: Item Wording**

The 9 items include from Hill & Willoughby’s (2005) Genderism & Transphobia Scale that were used to form a measure of discomfort with male femininity in the current study were:

GTS6 - I have teased a man because of his feminine appearance or behaviour

GTS7 - Men who crossdress for pleasure disgust me

GTS8 - Children should be encouraged to explore their masculinity and femininity (reversed)

GTS10 - Men who act like women should be ashamed of themselves.

GTS11 - Men who shave their legs are weird.

GTS17 - A man who dresses like a woman is a pervert

GTS19 - Feminine boys should be cured of their problems

GTS21 - Passive men are weak.

GTS25 - Feminine men make me feel uncomfortable

**Section 2: Additional Analyses**

Some readers may be more interested in one of our sets of predictor variables (i.e., homonegativity, hierarchical worldviews) than the other. For those readers, we also show, below, the results when femmephobia is considered as a predictor of anti-gay behavior with each of these two sets of predictors separately.

**Femmephobia and Homonegativity as Predictors of Anti-Gay Behavior**

Table S1 shows the analysis assessing femmephobia in conjunction with the measures of homonegativity. Both measures of homonegativity were positively associated with the dependent variable in the initial step, although only modern homonegativity was significant. Supporting H2a, when we added femmephobia in a second step, it remained a strong and significant positive predictor of anti-gay behavior, over and above the homonegativity measures. Also, note that femmephobia alone accounts for over twice the variance as the other two predictors together.

Unexpectedly, femmephobia became a slightly *stronger* predictor when the other variables were entered into the model (compare the *b* value of .16 to the value of .12 when femmephobia was considered on its own). This unusual pattern suggests the presence of one or more suppressor variables. Furthermore, adding femmephobia changed the nature of the association of the other predictor variables with the outcome variable. Modern homonegativity now had no association with the dependent variable, and old-fashioned homonegativity switched directions, now being significantly and *negatively* associated with the outcome variable. See the Discussion section of the main article for further discussion of this suppressor effect.

**Table S1**

*Femmephobia as a Predictor of Anti-gay Behavior, in Conjunction with Homonegativity Measures*

|  | Initial | | |  | Adding Femmephobia | | |
| --- | --- | --- | --- | --- | --- | --- | --- |
| Variable | β | *b* (se) | 95% CI |  | β | *b* (se) | 95% CI |
| Modern homonegativity | **.19** | **.06 (.02)** | **[.02, .10]** |  | .01 | .00 (.02) | [-.04, .05] |
| Old-fashioned homonegativity | .11 | .03 (.02) | [-.01, .08] |  | **-.18** | **-.05(.02)** | **[-.09, -.02]** |
| *R*^2^ | .08^*^ | | |  |  | | |
| Femmephobia |  | | |  | **.61** | **.16 (.02)** | **[.11, .20]** |
| Δ *R*^2^ |  | | |  | .17^*^ | | |
| Final *R*^2^ |  | | |  | .25^*^ | | |

*Note*. 95% CI = 95% bootstrapped confidence interval.

^*^ *p* < .001.

**Femmephobia and Hierarchical Worldviews as Predictors of Anti-Gay Behavior**

Table S2 shows a similar, albeit more muted, pattern when femmephobia is considered in conjunction with the measures of hierarchical worldviews. Each worldview is a significant and positive predictor on the first step. When adding femmephobia, it holds its strength as a predictor, remaining just as strongly associated with anti-gay behavior as it was when considered independently. Femmephobia accounts for as much variability in the outcome variable as the other three predictor variables put together.

Again, the other variables change the nature of their associations when femmephobia is added. Narcissism becomes non-significant, social dominance orientation remains significant but at half its former strength, and right-wing authoritarianism changes its direction of association with the outcome variable. Again, see the main Discussion section for potential explanations as to why other predictor variables change when femmephobia is added.

**Table S2**

*Femmephobia as a Predictor of Anti-gay Behavior, in Conjunction with Measures of Hierarchical Worldviews*

|  | Initial | | |  | Adding  Femmephobia | | |
| --- | --- | --- | --- | --- | --- | --- | --- |
| Variable | β | *b* (se) | 95% CI |  | β | *b* (se) | 95% CI |
| Social Dominance Orientation | **.29** | **.09 (.02)** | **[.05, .13]** |  | **.14** | **.04 (.01)** | **[.02, .07]** |
| Right-Wing Authoritarianism | **.10** | **.02 (.01)** | **[.00, .04]** |  | **-.15** | **-.03(.01)** | **[-.05, -.01]** |
| Narcissism | **.12** | **.01 (.01)** | **[.00, .02]** |  | .09 | .01(.01) | [-.00, .02] |
| *R*^2^ | .14^*^ | | |  |  | | |
| Femmephobia |  |  |  |  | **.50** | **.12 (.02)** | **[.09, .16]** |
| Δ *R*^2^ |  | | |  | .13^*^ | | |
| Final *R*^2^ |  | | |  | .27^*^ | | |

*Note*. 95% CI = 95% bootstrapped confidence interval.

^*^ *p* < .001.
